# Comparison of macular retinal thickness measurements using spectral-domain and swept-source optical coherence tomography in healthy eyes

**DOI:** 10.3389/fmed.2025.1529719

**Published:** 2025-01-31

**Authors:** Huan Wan, Zhaode Wu, Ziling Liu, Bo Qin

**Affiliations:** ^1^The Second Clinical Medical College of Jinan University, Shenzhen, China; ^2^Department of Ophthalmology, Shenzhen People’s Hospital, The Second Clinical Medical College of Jinan University, Shenzhen, Guangdong, China; ^3^Shenzhen Aier Eye Hospital, Aier Eye Hospital, Jinan University, Shenzhen, China

**Keywords:** optical coherence tomography (OCT), spectral-domain OCT (SD-OCT), swept-source OCT (SS-OCT), retinal thickness measurement, consistency analysis

## Abstract

**Aim:**

This study compares retinal thickness measurements in healthy eyes using one SD-OCT and two SS-OCT devices to assess differences and consistency for clinical application.

**Methods:**

Forty-eight eyes with a mean age of 28.15 ± 8.85 years were enrolled. Retinal thickness was measured using Heidelberg Spectralis SD-OCT, Svision VG200 SS-OCT, and TowardPi En Face SS-OCT. Normally distributed data were presented as mean ± SD; non-normal data as median (P25–P75). Intraclass correlation coefficients (ICC) and Bland–Altman analysis were used to assess agreement, with a 7 μm error threshold.

**Results:**

Significant differences were found between the three devices (*p* < 0.001). SD-OCT measurements were consistently lower than SS-OCT (*p* < 0.001), while the two SS-OCT devices showed no significant differences except in the nasal region (*p* = 0.006). ICC values between SD-OCT and SS-OCT devices were low (0.125–0.532), while SS-OCT devices showed better agreement (ICC: 0.369–0.922). Bland–Altman analysis found only 8.33% of SD-OCT and SS-OCT measurements within the 7 μm error range, compared to 81.25–83.33% for SS-OCT devices.

**Conclusion:**

The measurements of macular retinal thickness using SD-OCT and SS-OCT devices showed poor consistency and cannot be used interchangeably. However, measurements obtained from different SS-OCT devices demonstrated good consistency. To enhance the accuracy of results, it is recommended to maintain consistency in the devices used for follow-up examinations in the same patient.

## Introduction

Retinal thickness is a crucial parameter for diagnosing and assessing fundus diseases. Abnormal conditions such as edema, exudation, and hemorrhage in the retinal layers lead to an increase in retinal thickness, which is a key feature of ocular conditions such as age-related macular degeneration (AMD), diabetic retinopathy (DR), and central retinal vein occlusion (CRVO) (1–3). Therefore, changes in retinal thickness serve as indicators for evaluating the severity of fundus diseases and assessing the effectiveness of treatment. As treatments for retinal diseases continue to advance, reliable methods are needed to assess retinal thickness at different stages in both clinical practice and research. Optical coherence tomography (OCT) plays a vital role in detecting and evaluating fundus diseases (4). It offers several advantages, including being non-contact, non-invasive, and capable of providing high-resolution cross-sectional images of retinal layers quickly. OCT can vividly display abnormal retinal thickening, structural disruptions, subretinal fluid, intraretinal fluid, and retinal neovascularization. It holds significant value in the diagnosis, classification, prognosis, and treatment guidance of conditions such as AMD, choroidal neovascularization (CNV), and diabetic macular edema (DME) (3, 5, 6). With its automatic measurement of retinal thickness, OCT also provides a quantitative indicator for disease diagnosis and treatment evaluation. Recent treatment guidelines for AMD and DME have included OCT-derived biomarkers as essential tools for disease diagnosis and follow-up. The technology behind OCT has developed rapidly—from time-domain OCT (TD-OCT) in the 1990s to spectral-domain OCT (SD-OCT) in the 2000s, and now to swept-source OCT (SS-OCT). Each technological advancement has improved scanning speed, depth, field of view, morphological detail, and image quality. Today, various OCT devices, each with unique hardware configurations and software platforms, are widely used in clinical practice (7, 8).

However, standardized reference ranges for retinal thickness have yet to be established. Additionally, discrepancies and inconsistencies between measurements from different devices pose challenges, making it difficult for patients to undergo follow-up assessments seamlessly. Some studies have shown that SS-OCT and SD-OCT have good consistency and correlation in measuring central corneal thickness, corneal epithelial thickness and outer retinal thickness in healthy people (9–12). However, some studies have shown that the measurement values of SS-OCT and SD-OCT are not suitable for mutual conversion (13–15). Among the latest SS-OCT devices are the VG200 (a swept-source OCT by Svision) and the Ultrawide-field En Face OCT (by TowardPi), both offering high scanning speeds and superior resolution. However, no comparative studies have been conducted to evaluate the differences and consistency between these SS-OCT devices and SD-OCT in measuring macular retinal thickness.

**Figure 1:**
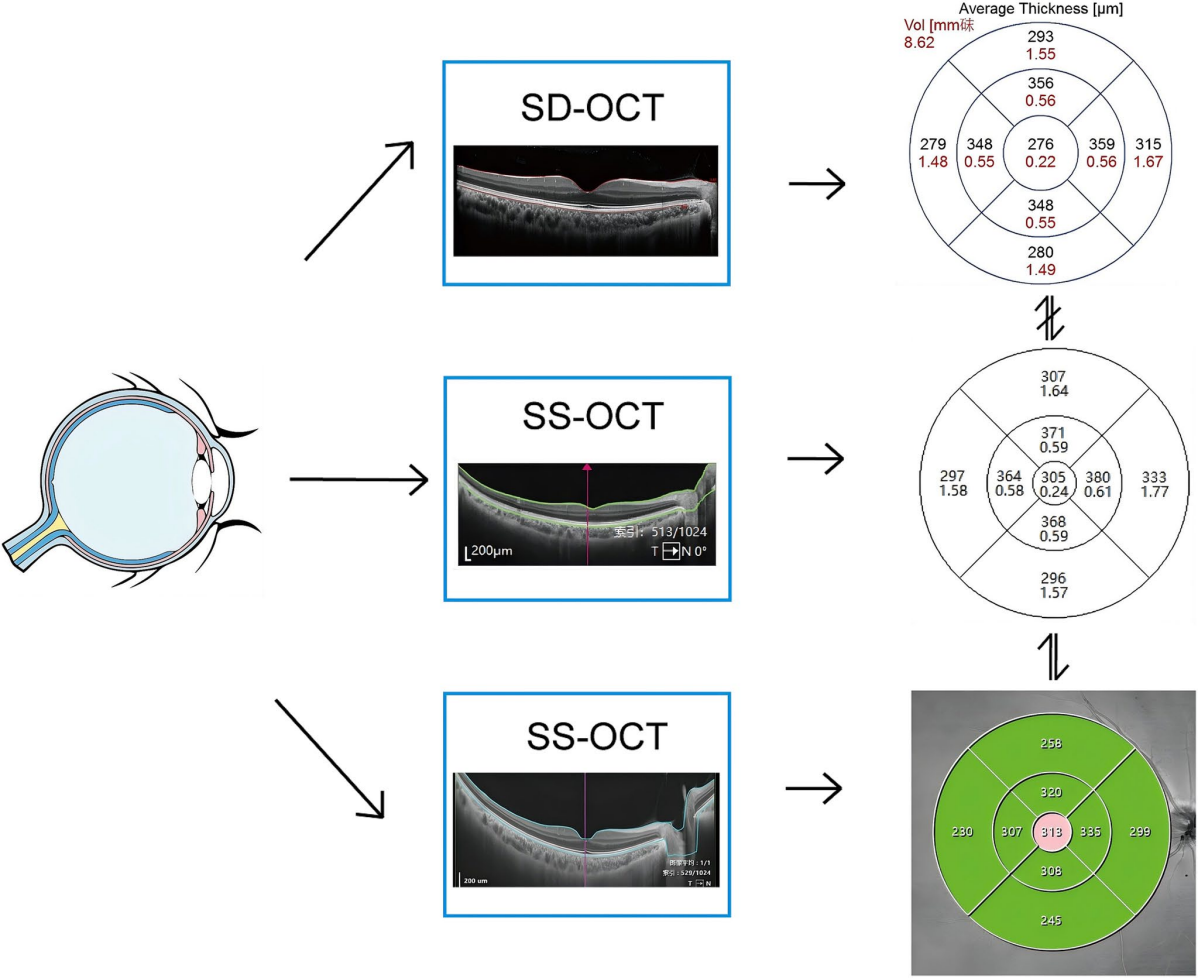
Schematic diagram of the experimental workflow.

This study aims to measure macular retinal thickness in healthy eyes using three OCT systems, Svision VG200 SS-OCT, TowardPi En Face SS-OCT and Heidelberg Spectralis SD-OCT. It will analyze the differences and consistency among the three instruments to provide evidence-based guidance for clinical treatment (Figure 1).

## Subjects and methods

### Ethical approval

Ethics approval was obtained from the Ethics Committee (2024-KY004-01) of Shenzhen Aier Eye Hospital, adhering to the tenets of the Declaration of Helsinki.

### Subjects

This study included 48 eyes examined at the Department of Ophthalmology, Shenzhen Aier Eye Hospital, in December 2023. The average age of participants was 28.15 ± 8.85 years (range: 7–51 years), with 18 eyes from males (37.5%). The mean spherical equivalent was−3.42 ± 2.29 D, and the best-corrected visual acuity (BCVA) was 1.04 ± 0.15.

### Inclusion and exclusion criteria for participants

The inclusion criteria were as follows: (1) uncorrected visual acuity (UCVA) >0.5; (2) cooperating with fixed vision; (3) voluntary participation for free testing using three OCT devices. The exclusion criteria included: (1) inability to cooperate with the examination; (2) previous retinal surgery; (3) retinal or choroidal diseases; (4) history of ocular hypertension or glaucoma; (5) use of ocular medication within the last 3 months.

### General examinations

All participants underwent UCVA testing, followed by Computer Optometry (CV-5000, Topcon, Japan). Medical histories were collected through questionnaires, and a slit-lamp microscope (SL-130, Carl Zeiss, Germany) was used to rule out other ocular conditions.

### Retinal thickness measurement

All retinal thickness measurements were performed on the same day for each participant by a single experienced examiner to ensure consistency. Three devices were used sequentially for both eyes: the Heidelberg Spectralis HRA + OCT, the VG200 Swept-Source OCT from SVision Imaging Ltd., and the Ultrawide-field En Face OCT from TowardPi Medical Technology Ltd. The Heidelberg device employed the dense scan mode (49 lines) to obtain macular thickness within a 6 mm diameter (automatic scan range 30°X25°). The VG200 Swept-Source OCT used a Cube 9 × 9, 1024 × 1024 scanning protocol to collect measurements from the same 6 mm macular area (automatic scan range 9 × 9 mm). The Ultrawide-field En Face OCT operated in 3D macular mode to gather data from the 6 mm macular area (automatic scan range 6 × 6 mm).

### Statistical analysis

All statistical analyses were performed using SPSS version 27.0 (IBM, United States). The Shapiro–Wilk test was used to check for normality. Normally distributed data were expressed as mean ± standard deviation (SD), and non-normally distributed data were expressed as median (P25–P75). Data following a normal distribution were analyzed using one-way analysis of variance (ANOVA) with repeated measures, while data not conforming to a normal distribution were analyzed using the Friedman *M*-test for multiple correlated samples. Intraclass correlation coefficients (ICC) assessed the agreement between devices, and Bland–Altman analysis evaluated the consistency of measurements. *p*-value <0.05 was considered statistically significant.

## Results

**Figure 2:**
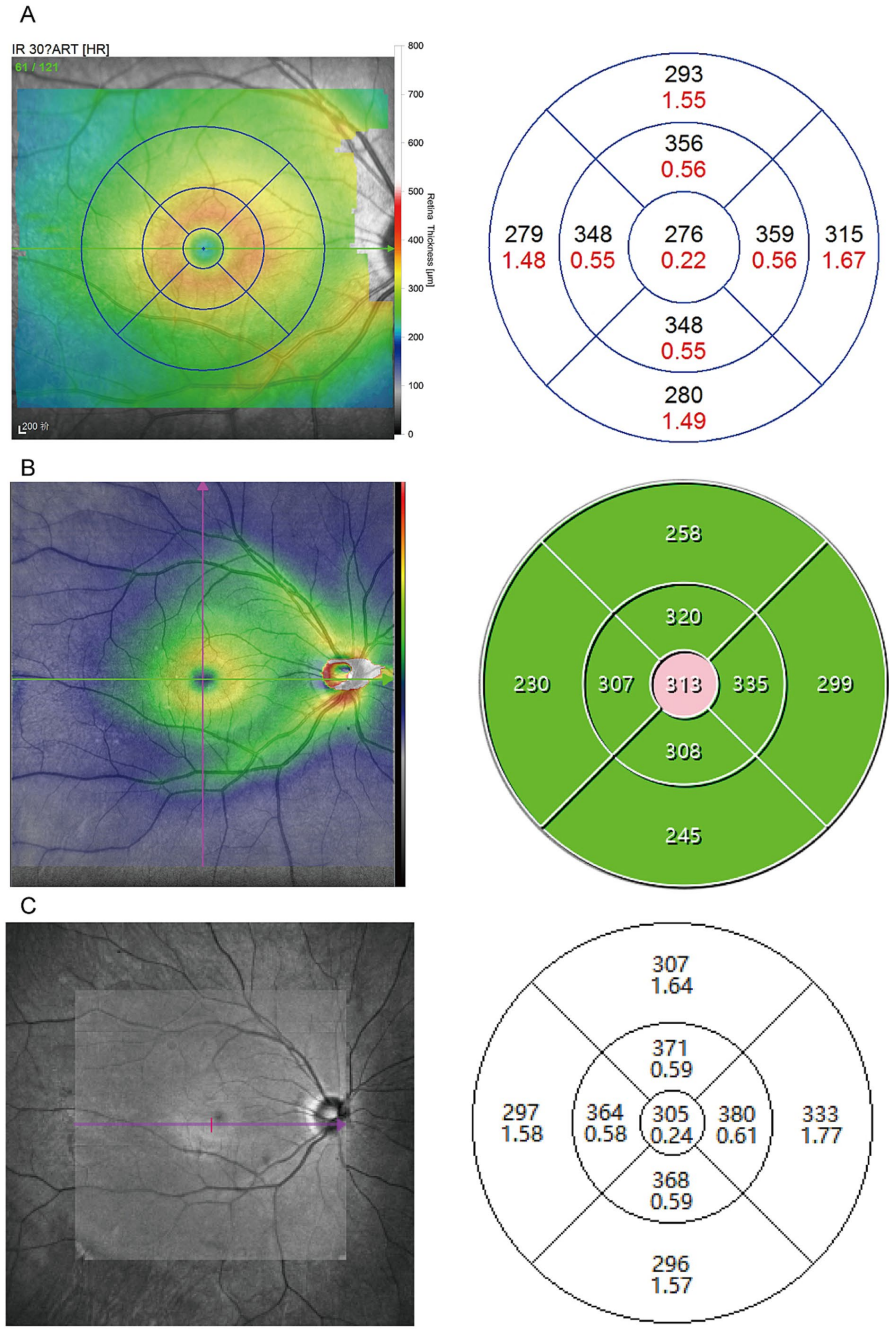
Sample scan using OCT deices from the SD-OCT and two SS-OCT. **(A)** Example case using Heidelberg device employed the dense scan mode (49 lines) to obtain macular thickness within a 6 mm diameter. **(B)** Example case using the VG200 Swept-Source OCT used a Cube 9 × 9, 1024 × 1024 scanning protocol to collect measurements from the same 6 mm macular area. **(C)** Example case using the Ultrawide-field En Face OCT operated in 3D macular mode to gather data from the same region.

A total of 48 eyes were included in the study, conducted in December 2023. The average age of the participants was 28.15 ± 8.85 years, with 18 males (37.50%). The mean spherical equivalent refraction was −3.42 ± 2.29 D, and the best-corrected visual acuity (BCVA) was 1.04 ± 0.15. Among them, three participants had a history of refractive surgery (Table 1). Figure 2 shows examples of three optical coherence tomography devices with different performances. The retinal thickness measurements obtained from the Heidelberg, SVision, and TowardPi devices for various retinal regions are summarized in Table 2. Data that followed a normal distribution (such as the central macular region and the nasal side within a 3 mm diameter) are presented as mean ± standard deviation. Non-normally distributed data are expressed as median (P25–P75).

**Table 1:** Baseline characteristics of study population.

Characteristics	*n*	Mean ± SD/*n* (%)
Age	48	28.15 ± 8.85
Gender (male)	48	18 (37.50%)
Spherical equivalent	48	−3.42 ± 2.29
Best corrected visual acuity	48	1.04 ± 0.15
History of eye surgery	48	3 (6.25%)

Numerical variables are represented by mean ± standard deviation, and categorical variables are represented by number of cases (%).

**Table 2:** Macular retinal thickness measured by different devices.

	Mean ± SD/median (P25–P75) (μm)		
Field	TowardPi	Heidelberg	SVision
Center	286.69 ± 17.70	255.81 ± 17.45	287.52 ± 18.18
Minimum	234.00 (228.00–239.00)	211.50 (243.50–271.75)	231.00 (220.50–239.00)
3 mm superior	364.61 ± 11.23	343.22 ± 11.96	363.00 (347.25–371.75)
3 mm inferior	365.65 ± 15.16	339.96 ± 15.08	359.13 ± 19.36
3 mm temporal	358.92 ± 12.89	339.57 ± 13.04	355.50 (335.25–365.75)
3 mm nasal	350.95 ± 12.66	329.45 ± 13.23	348.00 (331.50–354.75)
6 mm superior	325.32 ± 11.38	308.27 ± 10.84	321.00 (310.00–332.75)
6 mm nasal	346.50 (336.25–351.00)	324.06 ± 10.68	342.00 (332.25–350.75)
6 mm inferior	309.29 ± 18.25	293.24 ± 12.30	303.50 (286.00–315.00)
6 mm temporal	305.50 (300.25–314.75)	290.00 (281.25–301.25)	303.50 (287.25–310.00)

Variables that conform to the normal distribution are represented by the mean ± standard deviation, and variables that do not conform to the normal distribution are represented by the median (P25–P75).

The results indicated significant differences in retinal thickness measurements across all regions among the three devices (*p* < 0.001). Pairwise comparisons revealed significant differences between SD-OCT (Heidelberg) and either of the two SS-OCT devices (SVision and TowardPi) in all regions (*p* < 0.001). Notably, no significant differences were observed between the two SS-OCT devices (SVision and TowardPi) (*p* > 0.05) except in the nasal region within a 3 mm diameter (*p* = 0.006). The measurement data for the central macular region (within 1 mm) and the nasal side (within 3 mm) followed a normal distribution and were analyzed using repeated-measures ANOVA (Table 3). For regions where the data did not follow a normal distribution, including the minimum macular thickness and superior, inferior, and temporal regions within the 3 mm and superior, inferior, nasal, and temporal regions within the 6 mm diameters, the Friedman *M*-test for multiple related samples was used (Table 4).

**Table 3:** Difference and consistency of retinal thickness measured by one-way ANOVA.

Field	Devices	Mean difference (μm)	Std. error	*p*-value	ICC value	95% LoA	Within 95% LoA	Within ±7 μm
Center								
*F* = 421.682	Heidelberg-SVision	−31.715	1.478	<0.001	0.324	−51.78 to −11.65	93.75%	2.08%
*p* < 0.001	Heidelberg-TowardPi	−30.884	1.187	<0.001	0.350	−47.00 to −14.77	97.92%	0.00%
	SVision-TowardPi	0.831	1.028	1.000	0.922	−13.13 to 14.79	93.75%	83.33%
3 mm nasal								
*F* = 104.414	Heidelberg-SVision	−19.168	2.356	<0.001	0.348	−51.16 to 12.83	91.67%	8.33%
*p* < 0.001	Heidelberg-TowardPi	−25.691	0.865	<0.001	0.377	−37.43 to −13.95	93.75%	0.00%
	SVision-TowardPi	−6.524	1.987	0.006	0.645	−33.51 to 20.46	93.75%	79.17%

**Table 4:** The Friedman *M*-test for repeated measures design.

Field	Devices	Mean Difference (μm)	Standardized test statistic (*z*)	*p*-value	ICC value	95% LoA	Within 95% LoA	Within ±7 μm
Minimum								
*χ*^2^ = 79.948	Heidelberg-SVision	−17.01	−6.43	<0.001	0.499	−33.52 to −0.50	95.83%	12.50%
*p* < 0.001	Heidelberg-TowardPi	−19.06	7.348	<0.001	0.463	−30.70 to −7.42	95.83%	0.00%
	SVision-TowardPi	−2.05	0.919	1.000	0.902	−13.60 to 9.50	89.58%	81.25%
3 mm superior								
*χ*^2^ = 63.948	Heidelberg-SVision	−13.99	−5.001	<0.001	0.298	−46.49 to 18.51	93.75%	0.00%
*p* < 0.001	Heidelberg-TowardPi	−21.39	6.940	<0.001	0.349	−29.22 to −13.56	93.75%	0.00%
	SVision-TowardPi	−7.40	1.939	0.157	0.427	−37.72 to 22.92	91.67%	79.17%
3 mm inferior								
*χ*^2^ = 55.688	Heidelberg-SVision	−10.89	−4.491	<0.001	0.381	−46.77 to 24.99	93.75%	6.25%
*p* < 0.001	Heidelberg-TowardPi	−19.35	6.532	<0.001	0.423	−31.11 to −7.59	91.67%	4.17%
	SVision-TowardPi	−8.46	2.041	0.124	0.450	−42.65 to 25.72	93.75%	81.25%
3 mm temporal								
*χ*^2^ = 63.948	Heidelberg-SVision	−13.17	−5.001	<0.001	0.270	−49.41 to 23.06	93.75%	4.17%
*p* < 0.001	Heidelberg-TowardPi	−21.49	6.940	<0.001	0.369	−34.05 to −8.94	95.83%	2.08%
	SVision-TowardPi	−8.32	1.939	0.157	0.387	−41.77 to 25.12	93.75%	79.17%
6 mm superior								
*χ*^2^ = 61.455	Heidelberg-SVision	−6.77	−4.797	<0.001	0.359	−48.14 to 34.60	89.58%	0.00%
*p* < 0.001	Heidelberg-TowardPi	−17.04	6.838	<0.001	0.427	−25.32 to −8.76	100.00%	0.00%
	SVision-TowardPi	−10.28	2.041	0.124	0.369	−50.58 to 30.03	89.58%	79.17%
6 mm nasal								
*χ*^2^ = 71.273	Heidelberg-SVision	−17.58	−5.715	<0.001	0.346	−44.83 to 9.66	87.50%	8.33%
*p* < 0.001	Heidelberg-TowardPi	−22.05	7.144	<0.001	0.281	−41.96 to −2.13	93.75%	2.08%
	SVision-TowardPi	−4.47	1.429	0.459	0.780	−25.03 to 16.10	89.58%	83.33%
6 mm inferior								
*χ*^2^ = 50.234	Heidelberg-SVision	−4.45	−4.185	<0.001	0.425	−45.17 to 36.26	93.75%	8.33%
*p* < 0.001	Heidelberg-TowardPi	−14.44	6.226	<0.001	0.532	−27.49 to −1.39	93.75%	10.42%
	SVision-TowardPi	−9.99	2.041	0.124	0.433	−49.49 to 29.52	89.58%	79.17%
6 mm temporal								
*χ*^2^ = 45.091	Heidelberg-SVision	−2.52	−3.878	<0.001	0.161	−69.89 to 64.85	95.83%	8.33%
*p* < 0.001	Heidelberg-TowardPi	−15.52	5.920	<0.001	0.125	−63.05 to 32.00	95.83%	8.33%
	SVision-TowardPi	−13.00	2.041	0.124	0.440	−64.75 to 38.75	87.50%	81.25%

Intraclass correlation coefficient (ICC) analysis was performed to assess the agreement among the three devices. The correlations between Heidelberg and the two SS-OCT devices (SVision and TowardPi) were relatively poor, with ICC values ranging from 0.161 to 0.499 and 0.125 to 0.532, respectively. In contrast, the correlation between SVision and TowardPi was stronger, with ICC values ranging from 0.369 to 0.922 (Tables 3, 4).

**Figure 3:**
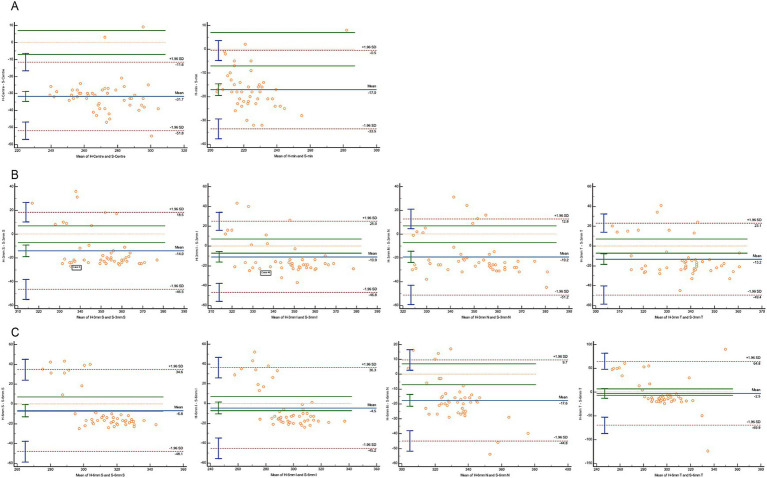
Bland–Altman plot for consistency between Heidelberg and SVision devices. **(A)** The central and minimum macular thickness between Heidelberg and SVision: the 95% limits of agreement (LoA) did not cross the zero line. **(B)** For four regions within the 3 mm diameters, the 95% LoA between Heidelberg and SVision crossed the zero line, with 91.67–93.75% of the points falling within the LoA. However, only 0.00–8.33% of the points fell within the allowable error range of 7 μm. **(C)** For four regions within the 6 mm diameters, the 95% LoA between Heidelberg and SVision crossed the zero line, with 87.50–93.75% of the points falling within the LoA. However, only 0.00–8.33% of the points fell within the allowable error range of 7 μm.

**Figure 4:**
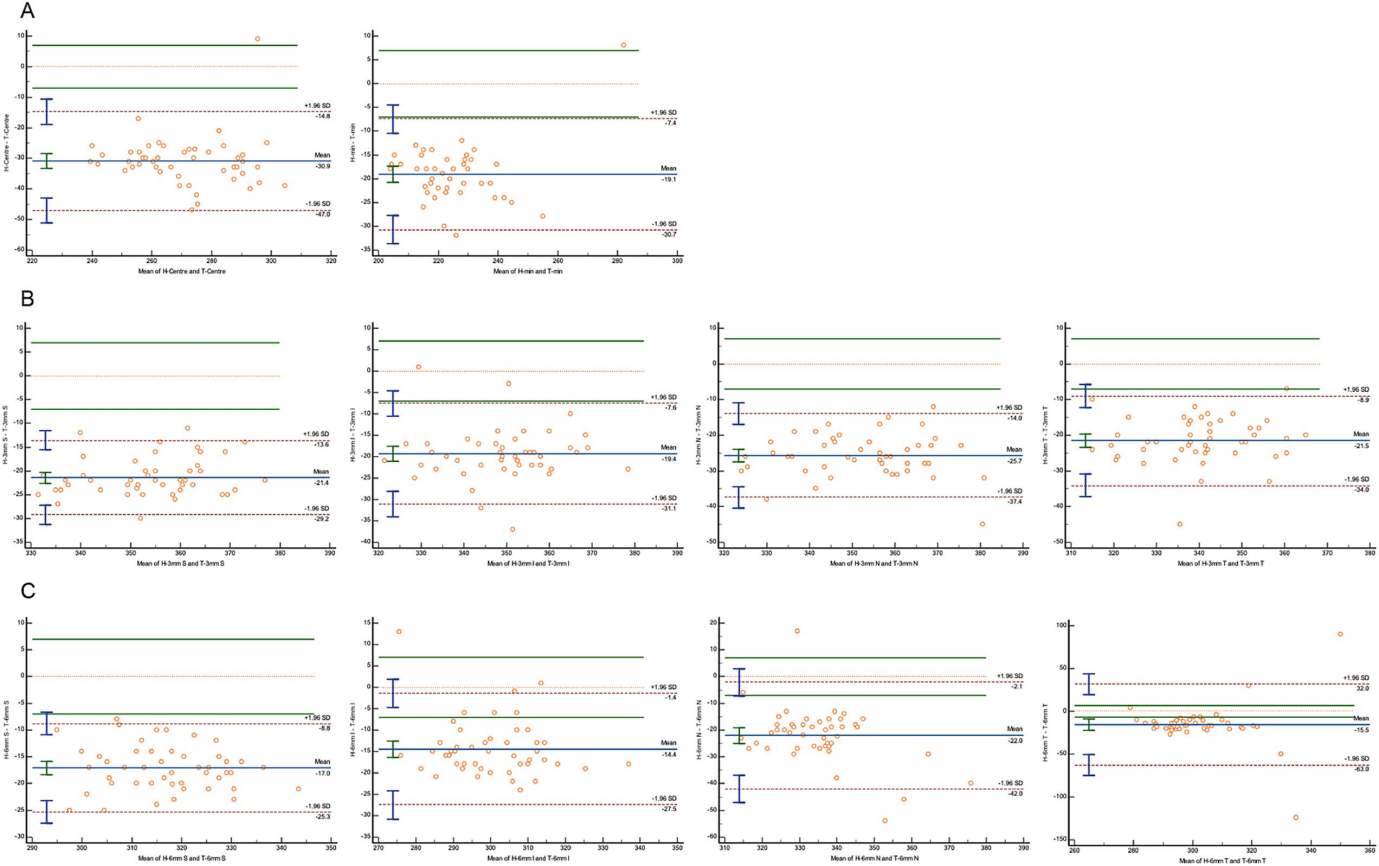
Bland–Altman plot for consistency between Heidelberg and TowardPi devices. **(A)** The central and minimum macular thickness between Heidelberg and TowardPi: the 95% limits of agreement (LoA) did not cross the zero line. **(B)** For four regions within the 3 mm diameters between Heidelberg and TowardPi: the 95% limits of agreement (LoA) did not cross the zero line. **(C)** For superior, inferior, nasal regions within the 6 mm diameters between Heidelberg and TowardPi: the 95% limits of agreement (LoA) did not cross the zero line. For temporal regions within the 6 mm diameters: the 95% LoA between Heidelberg and TowardPi is −63.05 to 32.00 (μm), 8.33% of the points fell within the allowable error range of 7 μm.

**Figure 5:**
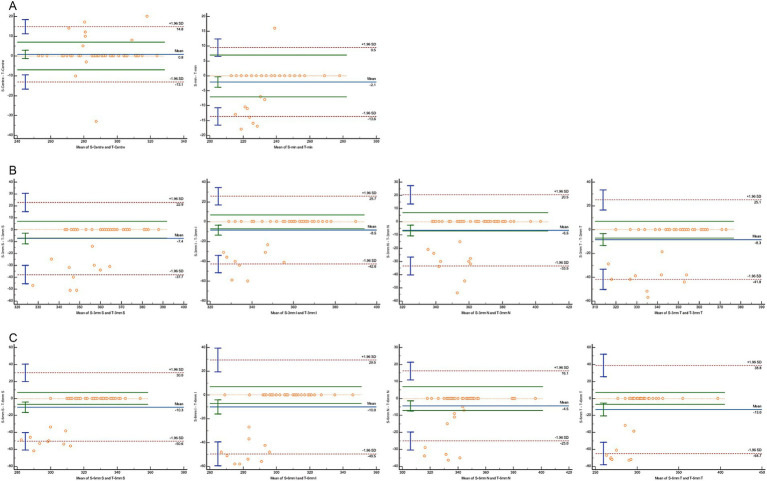
Bland–Altman plot for consistency between SVision and TowardPi devices. **(A)** The central and minimum macular thickness between SVision and TowardPi: the 95% limits of agreement (LoA) is −13.13 to 14.79 (μm) and −13.60 to 9.50 (μm), 83.33 and 81.25% of the points fell within the allowable error range of 7 μm. **(B)** For four regions within the 3 mm diameters between SVision and TowardPi: 93.75% of the points falling within the LoA, 79.17%–81.25% of the points fell within the allowable error range of 7 μm. **(C)** For four regions within the 6 mm diameters between SVision and TowardPi: 87.50%–89.58% of the points falling within the LoA, 79.17%–83.33% of the points fell within the allowable error range of 7 μm.

A Bland–Altman analysis was conducted to evaluate agreement, using 7 μm as the maximum allowable error (Figures 3–5). The 95% limits of agreement (LoA) for the central macular region and minimum macular thickness between Heidelberg and SVision did not cross the zero line, indicating significant systematic bias and poor agreement. For other regions within the 3 mm and 6 mm diameters, the 95% LoA between Heidelberg and SVision crossed the zero line, with 87.50–95.83% of the points falling within the LoA. However, only 0.00–8.33% of the points fell within the allowable error range of 7 μm (Figure 3). Similarly, for the temporal region within the 6 mm diameter, the 95% LoA between Heidelberg and TowardPi ranged from −63.05 to 32.00 μm, with 95.83% of the points falling within the LoA, but only 8.33% within the allowable error range. In other regions within the 6 mm diameter, the 95% LoA between Heidelberg and TowardPi did not cross the zero line, indicating significant systematic bias and poor agreement (Figure 4). In contrast, the 95% LoA between SVision and TowardPi crossed the zero line in all regions, with 87.50–93.75% of points falling within the LoA and 81.25–83.33% of points within the allowable error range (Figure 5).

## Discussion

With the rapid development of OCT technology, various OCT devices with different hardware and software have entered clinical practice. Understanding the consistency and discrepancies between spectral-domain OCT (SD-OCT) and swept-source OCT (SS-OCT) devices is essential for making accurate diagnostic conclusions and determining appropriate management strategies. SS-OCT has already been applied in the diagnosis and staging of conditions such as retinopathy of prematurity, diabetic retinopathy, age-related macular degeneration, and glaucoma, and it is expected to become the mainstream method for ophthalmic OCT in the future (16, 17). This study compared retinal thickness measurements of the fovea and surrounding macular region in healthy individuals using one SD-OCT device and two SS-OCT devices. Results indicated that retinal thickness measurements, a crucial parameter for tracking fundus diseases, showed poor consistency between SD-OCT and SS-OCT, meaning the measurements are not directly interchangeable. However, the two SS-OCT devices demonstrated good agreement, allowing their measurements to be cross-referenced in quantitative analyses.

The utilize of OCT represents a significant advancement in non-invasive macular imaging (18). OCT biomarkers have emerged as critical prognostic indicators for treatment outcomes in various diseases, such as diabetic macular edema (DME) (6). For a long period, SD-OCT has been regarded as the gold standard for visualizing retinal structural changes (19). It provides clear, high-resolution retinal imaging even in the presence of dense media opacities or small pupil sizes (20, 21). SS-OCT, with its faster scanning speed and longer wavelength, minimizes motion artifacts and provides better visualization of deeper retinal structures (15). However, one report indicates that SS-OCT may fail to detect approximately one-quarter of patients with actual macular disease, making it unsuitable as a replacement for SD-OCT in screening or diagnosing macular health (22).

The retinal thickness values obtained from the SD-OCT device were significantly lower than those from the SS-OCT devices, while the two SS-OCT devices showed no significant differences between each other. Intraclass correlation coefficient (ICC) analysis, which ranges from 0 to 1 (with values >0.75 indicating good reliability and <0.4 indicating poor reliability), revealed that SD-OCT measurements had poor reliability when compared to both SS-OCT (TowardPi and SVision) devices, with ICC values peaking at 0.499 and 0.532. In contrast, the SS-OCT devices (TowardPi and SVision) exhibited excellent reliability in the central macular region and minimum macular thickness, with ICC values of 0.922 and 0.902, respectively. Reliability for peripheral regions was moderate, with the lowest ICC value being 0.369 and the lowest ICC value being 0.369 and maximum value being 0.780. Using a 7 μm difference as the clinically significant allowable error (23), Bland–Altman analysis showed that only 8.33% of the measurements between the Heidelberg (SD-OCT) and the SS-OCT devices (SVision and TowardPi) fell within the allowable error range, indicating poor agreement. However, the measurements from the two SS-OCT devices demonstrated much better consistency, with 81.25–83.33% of the points within the allowable error range. These findings suggest that retinal thickness measurements from SD-OCT and SS-OCT devices are not directly interchangeable and should not be used as quantitative indicators during follow-up. However, SS-OCT devices (TowardPi and SVision) provided sufficiently similar measurements, allowing their results to be referenced against each other for clinical follow-up purposes.

The differences between SD-OCT and SS-OCT in image recognition and data measurement come from different hardware and software. From a hardware perspective, the Heidelberg Spectralis HRA + OCT, a widely used SD-OCT device, has demonstrated high stability and accuracy. SD-OCT typically acquires 27,000–40,000 A-scans per second with an axial resolution of approximately 3.5–6 μm (24). The layer-by-layer analysis of the retina provided by SD-OCT gives it a higher resolution than contact ophthalmoscopes and fundus photography (25). The VG200 swept-source OCT (SS-OCT) by SVision Imaging Ltd. (Luoyang, China) and the Ultrawide-field En Face OCT by TowardPi Medical Technology Ltd. (Beijing, China) operate on an SS-OCT-Angiography platform, enabling the simultaneous acquisition of structural and vascular parameters (26). SS-OCT technology uses a wide-spectrum light source to separate wavelengths over time and captures interference spectra with a high-speed single-point detector. This system achieves scanning speeds of up to 400,000 scans per second and a scanning depth of 6 mm, offering superior sensitivity decay characteristics, faster imaging speeds, and a broader imaging range (16, 27). The longer wavelength of SS-OCT reduces light scattering in the inner retina, improving the signal-to-noise ratio (13).

Different software systems used by OCT devices for image analysis further contribute to variations in defining the outer retinal layers (28, 29). The software used by the Heidelberg Spectralis HRA + OCT defined identify the most outer reflective band as the boundary of the outer retina (16). The Svision swept-source OCT defined Bruch’s membrane as the boundary of the outer retina while the TowardPi defined RPE (30, 31). In pathological conditions, where the RPE-Bruch’s membrane interface becomes more complex, measurement errors increase (32, 33). Accurate assessment of retinal thickness is critical for evaluating retinal edema, diagnosing retinal diseases, and selecting appropriate treatments, but these software differences can introduce variability in measurement outcomes.

In this study, the measurements obtained using SD-OCT and SS-OCT showed poor agreement, a phenomenon also reported in several other studies (22, 34). However, some articles have noted high consistency in measurements when using SD-OCT and SS-OCT devices from the same manufacturer (9). As SS-OCT becomes more widely applied in clinical practice, it is crucial for clinicians to consider the type of device used for diagnosis. Consistency in the device used during follow-up visits, matching the one used at the initial examination, is particularly important to ensure reliable results.

One limitation of this study is the use of different scan ranges and scan qualities across various OCT devices. Due to differences in scanning ranges, only the most representative retinal thickness measurements that were consistently detectable by all three devices could be selected, limiting the number of parameters available for comparative analysis. Scan quality is a factor that influences segmentation performance; thus, the highest quality scans were chosen on each device to minimize this effect. Additionally, some affected eyes were not included in the sample to assess the consistency of device measurements, and multiple measurements on the same patients were not conducted to evaluate the stability of the devices.

## Conclusion

In conclusion, the findings demonstrate significant systematic differences between SD-OCT and SS-OCT in macular retinal thickness measurements, rendering the values non-interchangeable. However, different SS-OCT devices showed good consistency in measuring macular retinal thickness, with values that are comparable across devices.

## Data Availability

The raw data supporting the conclusions of this article will be made available by the authors, without undue reservation.
